# Rationale and Evidence for Peripheral Nerve Stimulation for Treating Essential Tremor

**DOI:** 10.5334/tohm.685

**Published:** 2022-06-14

**Authors:** Aparna Wagle Shukla

**Affiliations:** 1 Department of Neurology, Fixel Institute for Neurological Diseases, University of Florida, Gainesville, Florida, United States of America

**Keywords:** Essential tremor, peripheral nerve stimulation, peripheral arm stimulation, sensory afferents, Cala system

## Abstract

**Background:**

There is growing recognition of peripheral stimulation techniques for controlling arm symptoms in essential tremor (ET). Recently, the FDA gave clearance to the Cala system, a device worn around the wrist to treat arm tremors. The Cala system stimulates the sensory afferents of the peripheral nerves with high-frequency pulses. These pulses are delivered to the median and radial nerves alternately at the tremor frequency of the individual patient.

**Methods:**

The PubMed database was searched using the terms (“Essential Tremor”[Mesh] OR “essential tremor” [Title/Abstract] OR “tremor” [Title/Abstract]) AND (“peripheral arm stimulation” [Title/Abstract] OR “Cala device” [Title/Abstract] OR “sensory afferent stimulation” [Title/Abstract] OR “afferent stimulation” [Title/Abstract] OR “arm stimulation” [Title/Abstract] OR “peripheral nerve stimulation” [Title/Abstract]).

**Results:**

The search yielded 54 articles. Many studies discussed the rationale and various strategies for peripheral modulation of tremor. While the Cala system was found to be safe and well-tolerated in ET, data on efficacy revealed mixed findings. In a large randomized, blinded trial (n = 77), the primary outcome evaluated with spiral drawing task did not improve but the secondary outcomes reflected by the arm tremor severity and the activities of the daily living score revealed 20–25% improvements. A subsequent trial (n = 323) found that the in-home use of the Cala device led to improvements of similar magnitude lasting for at least three months but the clinical assessments were open-labeled.

**Discussion:**

Peripheral stimulation techniques are promising therapeutic modalities for treating ET symptoms. Stimulation of sensory afferent nerve fibers at the wrist can potentially modulate the peripheral and central components of the tremor network. Although the Cala system is user-friendly, safe, and well-tolerated, the current clinical evidence on the efficacy is inconsistent and insufficient. Thus, more data is warranted for implementing peripheral nerve stimulation as a standard of care for ET.

**Highlights:**

The current review discusses the rationale, background, and potential mechanisms for using peripheral arm stimulation devices for treating ET. The Cala system is a wrist-worn peripheral nerve stimulation device that received FDA clearance to treat arm tremors. The current review evaluates the evidence for the safety and efficacy of using the Cala system and similar devices in clinical practice.

## Introduction

Essential tremor (ET) is the most prevalent movement disorder affecting about 1.3% of the global population of all ages [1]. Tremor can be functionally disabling and socially embarrassing as it interferes with many activities of daily living, leading to suboptimal quality of life [2,3,4,5]. While lifestyle modifications with weighted utensils and assistive writing devices can address mild tremor, pharmacotherapies are employed when the tremor becomes functionally disabling. Propranolol and primidone are the first-line medications effective in only 50% of patients even when titrated and tolerated to optimal doses [6]. Topiramate, alprazolam, clonazepam, and gabapentin that are next in line provide only 30–40% relief, and many patients may not tolerate these medications due to dose-limiting side effects [7,8]. The FDA-approved treatments such as deep brain stimulation (DBS) surgery [9] and focused ultrasound therapy are considered when the tremor shows refractoriness to oral medications [10,11]. These treatments are powerful as they directly target the pathogenic brain circuitry [12,13]. However, given the risks and limitations, surgical therapies are not recommended as first-line treatments [14]. DBS is invasive and costly, and some patients with long-term stimulation therapy develop tolerance to benefits [15]. Focused ultrasound therapy can lead to permanent clinical deficits as the ultrasound beam creates a lesion in the central brain circuitry. The long-term data for focused ultrasound therapy is not yet available. Thus, alternate, safe and effective treatments are warranted in the treatment armamentarium for ET.

Over the last two decades, there has been an emerging interest in using peripheral devices with electrical stimulators externally applied to the arm [16]. These noninvasive techniques can potentially control arm tremors by modulating the tremor circuitry at a peripheral level. A variety of peripheral stimulation devices have been tested and shown to have promising benefits. The FDA recently cleared the use of Cala system (Cala Health, Burlingame, CA, USA) in ET, a device worn at the wrist like a wristwatch. The current review discusses the rationale, background, and potential mechanisms for peripheral stimulation devices with a specific focus on the role of Cala system in ET.

## Methods

The PubMed database was searched using the terms (“Essential Tremor”[Mesh] OR “essential tremor” [Title/Abstract] OR “tremor” [Title/Abstract]) AND (“peripheral arm stimulation” [Title/Abstract] OR “Cala device” [Title/Abstract] OR “sensory afferent stimulation” [Title/Abstract] OR “afferent stimulation” [Title/Abstract] OR “arm stimulation” [Title/Abstract] OR “peripheral nerve stimulation” [Title/Abstract]). Filters included articles written in English and studies conducted on human subjects. Based on the screening of abstracts, 54 studies of interest were shortlisted. The bibliography of these articles was further searched to identify additional relevant articles.

### Rationale for peripheral nerve stimulation in ET

ET is thought to involve the cerebello-thalamo-cortical loop. The oscillations possibly originate in the synaptic organization of the Purkinje cells [17,18]. Although ET is central in origin; there is evidence to support a peripheral component in the pathophysiology, such as the mechanical properties of the arm, sensory feedback, and the sensorimotor reflex loop between the arm and the spinal cord [19,20,21]. While the mechanical factors have been found to mainly contribute to physiological tremor sensory feedback plays a crucial role in the pathogenesis of ET [22,23,24]. Previous studies found electrical stimulation of the median nerve evoked activity within the ventral intermedius nucleus (Vim) of the thalamus and other regions of the tremor network [25]. In one study, very-high-frequency oscillatory (VFO) activity in the range of 500 Hz was found to affect the activity recorded from the DBS contacts implanted within the Vim [25,26]. The VFOs were thought to be generated in the sensory nucleus of the thalamus, inducing time-locked firing of neurons within the Vim subregion [27]. Modulation of these peripherally generated activities could potentially decrease the tremor amplitude.

**Figure 1:**
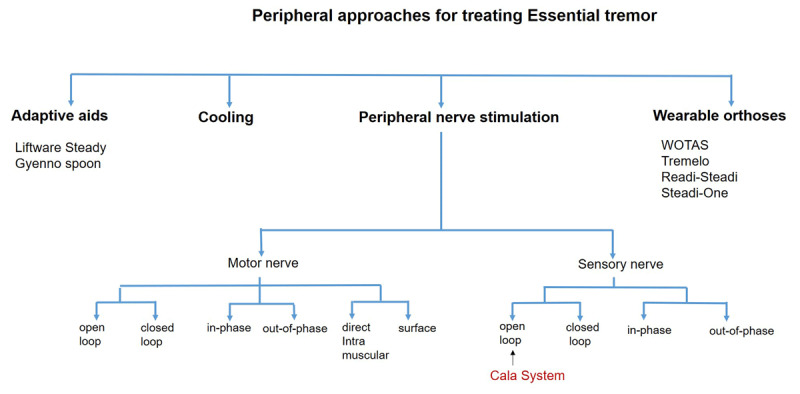
Peripheral interventions for treating essential tremor.

In a physiological assessment, limb weighting that involves adding weights to the tremoring arm can isolate the peripheral component from the central component. Limb weighting likely affects the biophysical properties of the peripheral stretch reflexes. In the accelerometric and surface EMG recordings, adding weights can lead to the generation of two frequency peaks; one associated with the peripheral component and the other related to the central component [28,29]. Besides limb weighting, limb cooling is another technique to modulate the tremor peripherally. Some studies found that surface cooling of the hand and forearm with an icepack or cold water can lower the tremor intensity [30,31]. Limb cooling presumably reduces the conduction speed of peripheral nerves or affects the properties of muscle spindles sending afferent information [30,32,33]. Thus, various methods can modulate the peripheral tremor component (Figure 1).

## Role of transcutaneous electrical nerve stimulation (TENS)

Transcutaneous electrical nerve stimulation (TENS) is a non-invasive peripheral stimulation technique developed primarily to relieve neuropathic pain [34]. TENS involves pulsed delivery of electrical currents to activate nerves underlying the intact skin [35]. TENS can be delivered to peripheral nerves innervating the forearm and the hands. The putative mechanism is a reduced transmission of noxious information through selective stimulation of large, myelinated A-beta fibers carrying touch and pressure sensations [34]. The technique is safe and can be self-applied through a battery-powered hand-held device. In theory, TENS modulates the sensory information contributing to the peripheral component of tremor.

Another similar technique is motor nerve stimulation is, commonly referred to as functional electrical stimulation (FES) [36,37,38]. Many tremor studies, in the beginning, focused on motor nerve stimulation instead of sensory information modulation. FES delivered in-phase would induce co-contraction of agonists and antagonists, and these would supposedly increase the impedance at the oscillating joints. FES delivered out-of-phase would lead to a generation of an antagonistic force to oppose the tremor-related activation of the agonist muscle [39]. Although FES was determined to be promising, important drawbacks such as muscle fatigue and patient discomfort led to the tempering of the enthusiasm [16]. Later studies experimented with stimulation of the sensory nerves instead of the motor nerves. Median and ulnar sensory stimulation at the wrist was observed to modulate the tremor frequency [40,41]. It was posited that stimulation of the sensory nerves increased the gain within the muscle spindle reflex loop leading to modulation of the tremor-like oscillations [42]. Sensory afferent stimulation can attenuate tremors without the undesirable side effects of muscle fatigue and discomfort associated with FES.

Some studies pursued closed-loop sensory stimulation to allow real-time adaption in response to kinematic data related to tremor. For example, in one study, gyro sensors were used to capture the angular velocity, peak power and peak frequency of tremor signals. These signals recorded from the finger, hand, and forearm joints triggered real time transcutaneous sensory stimulation lasting 15 seconds. The consequent effects were determined during and 5 minutes after stimulation [43]. Some studies employed electromyography to capture tremor signals for real time monitoring and stimulation [40,44,45].

## The Cala system

The Cala system is a user-friendly device worn around the wrist that stimulates the peripheral sensory nerves. The device has a detachable band customized to match the individual wrist circumference (small circumference: 13.5–15.4 cm, medium: 15.5–17.4 cm, large 17.5–19.5 cm). While specific preparation is not required, skin is recommended to be kept wet and free of lotion before applying the device. The Cala device should be worn on the more affected side. There are two hydrogel electrodes (2.2 cm × 2.2 cm) facing the median and radial nerves on the anterior surface of the wrist and one counter-electrode on the posterior surface of the wrist. The spacing between the electrodes varies according to wrist circumference (small, 1.3 cm; medium, 1.8 cm, and large, 2.3 cm). In the beginning, the device is calibrated according to the individual’s tremor frequency determined with the help of onboard accelerometers during a standard forward posture hold task. Stimulation delivered at a frequency of 150 Hz consists of a series of charge-balanced 300 μs biphasic pulses, with a 50 μs interpulse period between pulses. The median and radial nerves are stimulated alternately at a frequency equal to the tremor frequency of the individual. For example, for a 5 Hz tremor, continuous stimulation will be applied over the median nerve for 100 ms alternating with the radial nerve for 100 ms. The stimulation intensity is gradually escalated in 0.25 mA increments to monitor the paresthesia induced in the hand or finger area corresponding to distributions of the palmar digital branches of the median nerve and the superficial branch of the radial nerve. Final stimulation amplitude is the highest level of tolerable intensity reached. The device should be worn for 40 min before using the arm in activities of daily living the clinical benefits last around 90 min. The Cala system is not recommended in patients with implanted devices such as a pacemaker, defibrillator, or deep brain stimulator. Other contraindications for use include active seizure disorder, pregnancy, skin eruptions, open wounds, lesions, or infected skin areas.

### Clinical evidence for Cala system

The initial open-labeled studies with sensory afferent stimulation in ET revealed promising findings (Table 1). Subsequently, a small (n = 23) randomized controlled study employed a single 40-min long session of median and radial nerve stimulation with a benchtop (Digitimer, England) device [46]. Stimulation between median and radial nerves alternated as per the tremor frequency of the individual. The primary outcome was blinded rating of the Archimedes spiral task item of the Tremor Research Group Essential Tremor Rating Assessment Scale (TETRAS). The study found that the spiral drawing in the real-stimulation group improved significantly by about 60% compared to baseline, whereas the sham group did not reveal improvements (1.01 ± 0.22 vs. 0.37 ± 0.22; p < 0.05) [46]. These initial studies had limitations of practical applicability as they did not employ a user-friendly wearable system such as the Cala device.

**Table 1:** Clinical studies employing sensory afferent stimulation in ET.


STUDY	STUDY DESIGN	N	AGE IN YEARS	SEX (MALES)	STIM DEVICE	STIM LOCATION & STRATEGY	STIM DESIGN	STIM SETTINGS			STIM DURATION	OUTCOME MEASURES	TIMING OF OUTCOME ASSESSMENT	OUTCOME

								STIM AMPLITUDE	PULSE WIDTH	FREQUENCY IN HZ				

**Dosen et al 2013**	open labeled with objective physiology	2	72	Not avail	Axelgaard electrodes	wrist & fingers	closed loop	7.8 mA	300	100	2 sec	tremor power	real time	35%–48% reduction

**Heo et al 2015**	open labeled; physiology	18	68.8 ± 7.7	8	Cybermedic stimulator	arm & forearm	closed loop	0.2 mA	300	100	15 sec	angular velocity, peak power & frequency	real time & 5 min after	improvements in RMS & power with no change in frequency during and after

**Lin et al 2018**	randomized controlled; blinded & unblinded outcomes	23	70	11	Digitimer DS5	median & radial at wrist	open loop	5.9 ± 1.2 mA	300	150	single session; 40-min	TETRAS spiral item	immediately after	37% improvement in spiral

**Yu et al 2020**	open labeled; objective physiology	15	69.6 ± 10.2	9	Digitimer DS5	median & radial at wrist	open loop	not available	300	150	single session; 40-min	FTM-CRS & tremor power	real time, immediately after, 30 & 60min after	FTM-CRS score improved for 60min for 80% of patientsTremor power improved 60 min for 70% of patients.

**Kim et al 2020**	open labeled but study outcomes objective	9	67.6 ± 11.6	4	custom-built constant voltage mode stimulator	radial at wrist	open & closed loop	3.6 – 17.3 V	200	50, 100, 200	single session; 9 trials; 10-sec each	tremor power & peak frequency, qualitative assessment	real time monitoring	

**Pascual-Valdunciel et al 2020**	blinded & objective assessment	9	70.3	5	intramuscular thin film multichannel & surface Axelgaard stimulation	wrist flexors & extensors; intramuscular & surface stimulation	open & closed loop (out-of-phase & in-phase	2.4mA intramuscular; 5mA surface	200	100	30-sec each trial	Kinematics of wrist, elbow, shoulder & FTM-CRS	immediately after & 24 hrs after	closed loop intramuscular stimulation led to > 30% tremor reduction during & after; 4 patients 24 hr benefits


The Cala ONE device was examined for safety and efficacy in a randomized sham-controlled trial (n = 77) involving ET patients. (Table 2) In this pivotal trial, the primary outcome was the blinded rating of the TETRAS spiral item. With a single 40-min long stimulation session the spiral drawing did not improve significantly (p = 0.26). However, the secondary outcomes such as the blinded rating of Bain and Findley activities of daily living (ADL) items (42% vs. 28%; p = 0.001) and the unblinded scoring of the TETRAS upper limb item (49% vs. 27%; p = 0.017) revealed significant improvements [47]. A subsequent open-labeled PROSPECT trial that recruited 265 patients from 26 centers across North America assessed the longitudinal and long-term applicability of in-home use for the Cala Two device. Participants in the trial used the Cala device twice daily for three months [48]. The co-primary outcomes of the trial evaluated at one, two, and three months after stimulation were the clinician-rated TETRAS and patient-rated Bain and Findley ADL scale dominant hand scores. The secondary outcome was the accelerometer-based physiological assessment of tremor power. With a nearly 22% dropout, the study found significant improvements in the TETRAS dominant hand score and Bain and Findley ADL dominant hand scores at all follow-ups (p < 0.0001) [48]. After three months of the device use, there were 22% improvements in the TETRAS assessment and 28% improvements in the Bain and Findley ADL scores. The physiological data correlated significantly with the clinical ratings and there was a 50% reduction in tremor amplitude in nearly 54% of patients [48]. The investigators found that even though the magnitude of improvements on the TETRAS total score varied between patients, 62% of patients determined to have a “moderate to severe” tremor improved to a “mild” tremor category. Around 20% of patients reported skin irritations such as redness, itchiness, swelling, soreness or lesions that resolved using a topical ointment. Some patients complained of a stinging sensation, weakness, or burns that responded to a decrease in stimulation intensities. The adverse events were primarily mild to moderate in severity; overall, patients tolerated the device well [48].

**Table 2:** Clinical studies employing Cala device in ET.


STUDY	STUDY DESIGN	N ENROLLED	AGE IN YEARS	SEX (MALES)	STIM DEVICE	STIM LOCATION & STRATEGY	STIM DESIGN	STIM SETTINGS			STIM DURATION	OUTCOME MEASURES	TIMING OF OUTCOME ASSESSMENT	OUTCOME

								STIM AMPLITUDE	PULSE WIDTH	FREQUENCY IN HZ				

**Pahwa et al 2018**	randomized controlled; blinded and unblinded outcomes	93 (77 completed)	70.2 ± 10.6	45	Cala - one	median & radial sensory at wrist	open loop	5.4 ± 2.9 mA (average)	300	150	single session; 40-min	Primary: TETRAS spiral item; Secondary: TETRAS upper limb subscore, ADL and CGI-I	immediately after	Spiral score did not improve; upper limb tremor score improved (42 vs 28%), subject rated ADL scores improved (49 vs 27%)

**Isaacson et al 2020**	open labeled; outcomes also included objective physiology	263 (205 completed)	72.2 ± 8.6	126	Cala - two	median & radial sensory at wrist	open loop	not available	300	150	twice daily; 40-min per session; 3 months	Primary: TETRAS upper limb subscore, ADL & CGI-I; Secondary: tremor power	immediately after, at monthly in-clinic follow-up visits & at three months for long-term	Patients rated “Severe” or “Moderate” improved from 49.3% (TETRAS) and 64.8% (BF-ADL) at visit 1 pre-stimulation visit to 21.0% (TETRAS) and 23.0% (BF-ADL) at visit 3 post-stimulation and 54% had > 50% tremor reduction


The American Academy of Neurology follows a rigorous process rooted in evidence-based medicine methodology to review the evidence for an intervention efficacy. The risk of bias is measured using a four-tiered classification scheme with studies rated Class I are judged to have a low risk of bias, Class II is judged to have a moderate risk of bias, Class III, a moderately high risk of bias; and Class IV, a very high risk of bias. Based on the Class of evidence, adequacy of power, and consistency, practice recommendations with levels of certainty are formulated. Level A, the strongest level of recommendation, is employed if conclusive data is available from two Class I studies. Level B is the next level of recommendation if there is data from one Class I or two Class II studies. Level C is a recommendation with lower confidence level when data is available from one Class II study or two Class III studies. Level U indicates that the available evidence is insufficient to support or refute the efficacy of an intervention.

Currently, the efficacy data for the Cala system is available from a single open-labeled (Class IV) and a single randomized clinical trial (Class II). As evident in the results of the randomized clinical trial the primary outcomes did not reveal a significant change but the secondary analysis of blinded ADL ratings by patients and the unblinded upper limb tremor ratings by clinicians revealed significant improvements indicating an inconsistency in support of efficacy. Based on this efficacy data, there is currently insufficient evidence (level U) to support the use of the Cala device for control of the ET symptoms. Future studies with robust designs and conclusive evidence could lead to upgrading the practice level of recommendation. Nevertheless, the FDA has provided a Class II Medical Device clearance status, which means the manufacturer has shown that Cala is “substantially equivalent to another (similar) legally marketed device” that already has FDA clearance or approval. The Cala device, currently available only in the United States, is costly (around 3200 US dollars; with a return policy if ineffective), lacks the insurance coverage, and cannot be prescribed to patients with a DBS system. However, the clinical community could leverage the user-friendly nature and the established safety data to augment benefits from pharmacological therapies in ET.

### Potential mechanisms underlying clinical benefits with Cala device

In an 18F-fluorodeoxyglucose PET/CT study involving five ET patients, brain metabolism was measured at baseline and after 90 days following 40-min open-labeled stimulation sessions employed twice daily using the Cala device [49]. Tremor power and frequency were measured using an onboard three-axis accelerometer before and after all the transcutaneous afferent patterned stimulation was completed. Following 90 days of stimulation, the FDG PET/CT revealed increased metabolism in the ipsilateral and decreased metabolism in the contralateral cerebellar hemisphere. The pre-post kinematic measurement decreased tremor power, but there was no change in the tremor frequency [49].

As the cerebellum is a key pathogenic node in the ET network, these changes in metabolism were offered as the potential underlying mechanism to explain benefits from the Cala device. The authors speculated that an increased glucose utilization in the ipsilateral cerebellar cortex was related to alteration of Purkinje cell activity [49]. The study also found a decrease in metabolism of the ipsilateral pre- and post- central areas, occipital lobe, insula, cuneus, anterior cingulate, and inferior parietal cortex which cannot be attributed to functioning of tremor network. Furthermore, the study did not examine the relationship between brain metabolism change and tremors. Thus, these findings from a small open-labeled study sample that did not correct for multiple statistical comparisons cannot be interpreted with certainty. Further investigations are warranted to understand the brain adaptation response relevant to ET pathophysiology.

**Figure 2:**
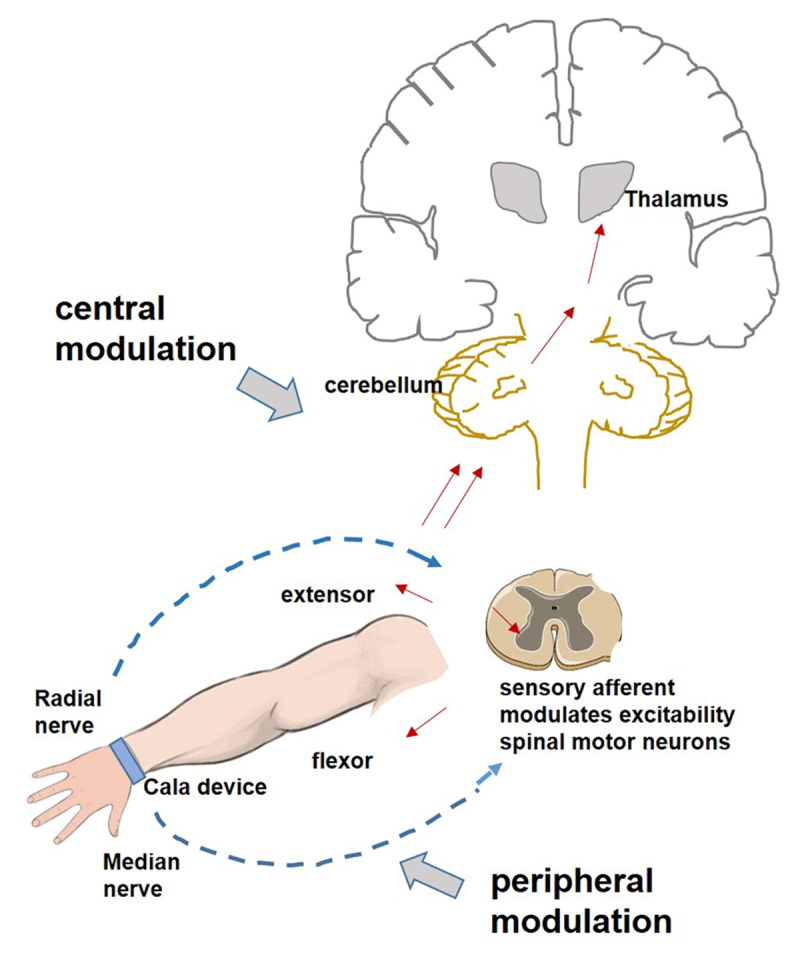
Possible peripheral and central mechanisms underlying the use of Cala device. In the peripheral mechanism, sensory afferents with median and radial nerve stimulation relayed to the spinal cord modulate the excitability of spinal motor neurons. Median nerve afferents increase flexor motor neuron excitability and radial nerve afferents increase extensor neuron excitability. Pattern of tremor bursts and phase and timing of afferent stimulation whether in-phase or out-of-phase will be important for control of tremor amplitude.

Another potential mechanism for Cala therapy is the peripheral modulation of tremor oscillations. The Cala device alternately activates the sensory afferent fibers of the median and radial nerves. The A-alpha sensory afferent stimulation supposedly carries the proprioceptive information from the muscle spindles and the Golgi tendon organs, leading to an increase in the excitability of the agonist spinal motor neurons and decrease in the excitability of antagonist spinal motor neurons [50]. When the agonist and antagonist muscles co-contract, the impedance at the oscillating joints increases, and when they contract out of the phase, the oscillating movements receive counteractive forces. Thus, the net result of the afferent input from the median nerve will activate the wrist flexors, and the radial nerve will activate wrist extensors. Whether the intrinsic pattern of tremor discharge is alternating or synchronous and whether the afferents are stimulated in-phase or out-of-phase will determine the final effects. These potential peripheral mechanisms have not been examined yet (Figure 2).

### Other devices & techniques that could be applied peripherally

Prochazka and colleagues around the 1990s first pioneered FES that directly triggers muscle contractions of the peripheral arms. FES was observed to result in nearly 70% tremor improvement [36,51]. Although direct intramuscular stimulation could lead to more significant tremor suppression, practically, it may not be viable. Thus, transcutaneous FES is more appealing and has shown promising data in multiple small cohort studies. Some studies have used real-time monitoring with EMG-based sensing algorithms to allow precise delivery of electrical pulses to the muscle stimulation [51]. A few devices for potential in-home use include the TREMOR neurorobot and a special glove adopting a co-contraction stimulation strategy to increase the stiffness of the limb via continuous stimulation of antagonistic flexor and extensor muscles of the arm [16]. On the other hand, the MOTIMOVE system (Belgrade, Serbia) with CE Marketing consists of a multichannel stimulator for out-of-phase stimulation [38]. FES commonly leads to side effects such as hand numbness, burning sensation, and muscle fatigue due to continuous active muscle contraction and joint activation. These side effects are essential as they can potentially limit long-term practical use.

Wearable orthotic systems are another group of devices for peripheral control of tremor [52]. These devices work on the principles of active force generation to counteract the involuntary movement related to tremor or passively suppress the oscillatory movements through dissipation of energy [16]. While the active systems may not be practical for day to day use as they are bulky, the passive systems such as the Viscous Beam system, Tremelo (Five Microns, Fresno, CA, USA), Steadi-One (Steadiwear, Toronto, ON, Canada), and Readi-Steadi (Readi-Steadi, Gonzales, LA, USA) are promising but do not have the aesthetic advantages of Cala [16]. Furthermore, safety and efficacy data from large sham-controlled randomized trials are currently lacking.

## Summary

The current pharmacological and surgical therapies primarily target the central tremor networks in ET. Emerging evidence indicates that the peripheral stimulation technique can also control the arm tremor. The FDA recently cleared the Cala system that achieves peripheral arm stimulation via a safe wearable device worn around the wrist. Cala device alternates pulses to the median and radial nerve, stimulating large, afferent myelinated fibers at a frequency equal to the tremor frequency. Recent clinical trials in ET found that Cala is safe and well-tolerated. Although the device available in the United States is user-friendly, many patients cannot afford it because insurance companies do not cover the costs. The current data for efficacy from clinical trials is inconsistent and inconclusive. More clinical research and experience will be needed for future consideration of Cala therapy and other similar systems as a standard of care for patients with ET.

## Financial Disclosure

AWS reports grants from the NIH and has received grant support from Benign Essential Blepharospasm Research foundation, Dystonia coalition, Dystonia Medical Research foundation, National Organization for Rare Disorders and grant support from NIH (KL2 and K23 NS092957-01A1) as a PI. She receives support from NIH Ro1 NS121120-01 as a Co-I. AWS has received consultant fees from Merz, Jazz and Acadia. She is the current Vice President for the Tremor Research Group and advisor for Biogen.
